# Impaired macrophage phagocytosis of bacteria in severe asthma

**DOI:** 10.1186/1465-9921-15-72

**Published:** 2014-06-27

**Authors:** Zhike Liang, Qingling Zhang, Catherine MR Thomas, Kirandeep K Chana, David Gibeon, Peter J Barnes, Kian Fan Chung, Pankaj K Bhavsar, Louise E Donnelly

**Affiliations:** 1 Airway Disease, National Heart and Lung Institute, Imperial College London, & Biomedical Research Unit, Royal Brompton & Harefield NHS Trust, London SW3, UK; 2 State Key Laboratory of Respiratory Diseases, The First Affiliated Hospital & Guangzhou First People’s Hospital, Guangzhou Medical University, Guangzhou, China; 3 National Heart & Lung Institute, Dovehouse St, London SW3 6LY, UK

**Keywords:** Macrophages, Phagocytosis, *Staphylococcus aureus*, *Haemophilus influenzae*, Asthma

## Abstract

**Background:**

Bacteria are frequently cultured from sputum samples of severe asthma patients suggesting a defect in bacterial clearance from the airway. We measured the capacity of macrophages from patients with asthma to phagocytose bacteria.

**Methods:**

Phagocytosis of fluorescently-labelled polystyrene beads, *Haemophilus influenzae* or *Staphylococcus aureus* by broncholaveolar lavage alveolar macrophages (AM) and by monocyte-derived macrophages (MDM) from non-asthmatics, mild-moderate and severe asthmatic patients was assessed using fluorimetry.

**Results:**

There were no differences in phagocytosis of polystyrene beads by AMs or MDMs from any of the subject groups. There was reduced phagocytosis of *Haemophilus influenzae* and *Staphylococcus aureus* in MDMs from patients with severe asthma compared to non-severe asthma (p < 0.05 and p < 0.01, respectively) and healthy subjects (p < 0.01*and* p < 0.001, respectively). Phagocytosis of *Haemophilus influenzae* and *Staphylococcus aureus* by AM was also reduced in severe asthma compared to normal subjects (p < 0.05). Dexamethasone and formoterol did not suppress phagocytosis of bacteria by MDMs from any of the groups.

**Conclusions:**

Persistence of bacteria in the lower airways may result partly from a reduced phagocytic capacity of macrophages for bacteria. This may contribute to increased exacerbations, airway colonization and persistence of inflammation.

## Introduction

Patients with asthma are usually well-controlled with inhaled corticosteroids (CS) and long-acting β_2_-agonists, but a proportion of patients, described as severe asthmatics, continue to experience uncontrolled asthma in spite of these treatments [1]. These patients consume a significant proportion of medical resources in terms of pharmacological treatments, hospital admissions or use of emergency services, and time off work or school [2]. Respiratory infections are often associated with wheezing episodes and may also impact on the development and severity of asthma. Viral and bacterial infections or colonization with bacteria could lead to chronic lower airway inflammation, impaired mucociliary clearance, increased mucus production and worsening of asthma [3,4]. Both viral and bacterial infections have been recognized as drivers of asthma exacerbations [5]. Various bacterial species have been cultured from sputum samples of patients during exacerbations [6], and also during a stable period in moderate-to-severe asthma patients [7,8]. Gram-positive *Staphylococcus aureus* and Gram-negative *Haemophilus influenzae* were two of the most common bacterial species cultured from induced sputum of patients with severe asthma [8]. The more sensitive technique of 16S ribosomal RNA microarray to detect bacterial species in the lower airways has revealed an increase in bacterial burden and diversity in patients with mild-to-moderate asthma compared to non-asthmatic individuals [9,10]. Thus, there appears to be an increased propensity for asthmatic subjects to carry more bacterial pathogens in their lower airways.

Macrophages produce a variety of cytokines and mediators that are vital for immune and inflammatory responses in their response with external agents. Macrophages are also important for the removal of particulates and bacteria from the airways [11]. This removal of potentially pathogenic micro-organisms via phagocytosis is essential for maintaining a non-pathogenic environment within the lung. However, the phagocytic capacity of macrophages for bacteria may be impaired in various pulmonary conditions. Thus, lung macrophages from children with severe asthma showed reduced phagocytic responses to *Staphylococcus aureus*[12]. Similarly, defective efferocytosis by AMs from severe asthmatics but not from mild-moderate asthmatics has also been reported [13]. This defect has been postulated to underlie the chronic inflammatory state due to the accumulation of bacteria and apoptotic and necrotic cells in the airways. In chronic obstructive pulmonary disease (COPD), a number of studies have shown defects in both bacterial clearance [14,15] and efferocytosis [16] indicating impaired macrophage innate responses.

We hypothesize that alveolar macrophages (AMs) and macrophages derived from blood monocytes (MDMs) from subjects with asthma may show impaired phagocytosis of *Haemophilus influenzae* and *Staphlycoccus aureus* when compared to non-asthmatic subjects. In addition, we examined the potential effects of the asthma treatments, corticosteroids and β-adrenergic agonists, on these phagocytic responses *in-vitro*.

## Methods

### Study participants

Patients with asthma were recruited from the Asthma Clinic of the Royal Brompton Hospital, London. All patients demonstrated either an improvement in FEV_1_ of >12% baseline FEV_1_ after inhaling 400 μg salbutamol aerosol or bronchial hyper-responsiveness defined by a concentration of methacholine provoking a fall in FEV_1_ of 20% or more (PC_20_) of <8 mg/ml. Current and ex-smokers of >5 pack-years were excluded. Patients on steroid-sparing agents such as methotrexate, those with a concurrent diagnosis of clinical bronchiectasis or with a respiratory tract infection requiring antibiotic treatment within 6 weeks of enrolment were excluded.

Severe asthmatics were defined according to the American Thoracic Society’s major criteria of needing either continuous or near-continuous oral corticosteroids or high dose inhaled corticosteroids (2,000 μg beclomethasone-equivalent per day or more) or both in order to achieve a level of mild-moderate persistent asthma, and also by the presence of 2 or more minor criteria of asthma control [17]. Patients with non-severe asthma were those who did not fall into the severe asthma category and who used up to 1000 μg inhaled beclomethasone or equivalent dosage per day with well-controlled asthma. Healthy volunteers with no diagnosis of asthma and with a negative PC_20_ (>16 mg/ml) were also recruited. The study protocol was approved by the Ethics Committee of Royal Brompton & Harefield NHS Trust/National Heart & Lung Institute, London UK. All volunteers gave their written informed consent.

### Monocyte isolation and MDM differentiation

Peripheral blood mononuclear cells (PBMCs) were isolated from 80 ml venous blood using Ficoll-Hypaque-Plus. PBMCs and re-suspended in MDM complete media (RPMI-1640 supplemented with 10% (v/v) FCS, 100 U/ml penicillin, 100 μg/ml streptomycin, 2 mM L-glutamine), at 1 × 10^6^ cells/ml and seeded on 96-well black plates (10^5^ cells/well) for 2 h at 37°C, 5% (v/v) CO_2_ to allow monocytes to adhere to the plate. Non-adherent cells were aspirated and monocytes were incubated with fresh complete media containing GM-CSF (2 ng/ml; R&D Systems, Abingdon, UK). Monocytes were incubated at 37°C, 5% (v/v) CO_2_ for 12 days to allow full differentiation into MDMs; fresh media containing GM-CSF were replenished on days 4 and 7 [15,18,19].

### Fibreoptic bronchoscopy and alveolar macrophage isolation

Fibreoptic bronchoscopy was performed using topical anaesthesia with lignocaine to the upper and lower airways under conscious sedation with intravenous midazolam. Warmed 0.9% (w/v) NaCl solution (50 ml × 4) was instilled into the right middle lobe and bronchoalveolar lavage (BAL) fluid was recovered by gentle hand suction. BAL fluid was centrifuged (400 g for 10 min) and the resultant cell pellet washed with Hanks’ balanced salt solution (HBSS). Cells were then suspended in culture media (RPMI-1640, containing 10% (v/v) foetal calf serum (FCS), 100U/ml penicillin, 100 μg/ml streptomycin, 2.5 μg/ml amphotericin, 2 mM L-glutamine). Cytospins were prepared and stained with Diff Quick stain (Harleco, Gibbstown, NJ, USA) for differential cell counts. Alveolar macrophages (AMs) were purified by adhesion to the plastic well for 4 h and then cultured overnight prior to experimentation.

### Phagocytosis assay

Phagocytosis was measured as described previously [15]. AMs and MDMs were exposed, for 4 h, to fluorescently-labelled polystyrene beads (2 μM diameter) at a concentration of 50 × 10^6^ beads/ml or to heat-killed non-typeable *H. influenzae* or to *S. aureus,* all labelled with Alexa-Fluor 488 conjugate (Invitrogen, Paisley, Scotland). AMs and MDMs were washed with Dulbecco-phosphate buffered saline and extracellular fluorescence was quenched with 1% (w/v) trypan blue at room-temperature. Trypan blue was aspirated and phagocytosis of fluorescent-labeled beads and bacteria by AMs or MDMs was measured using a Fluostar Optima fluorimeter (BMG LabTech, Aylesbury, Buckinghamshire) at excitation wavelength of 480 nm and emission wavelength of 520 nm. Data were expressed as relative fluorescent units (RFU). In order to determine that the beads or bacteria were being internalised, cells were exposed to cytochalasin D (5 μg/ml) for 30 min, prior to incubation with bacteria.

Confocal microscopy was used to visualize whether the beads or bacteria were internalised. MDM (2×10^5^cells) were cultured on well Lab-tek Permanox chamber slides and phagocytosis performed as described above. Cells were then incubated at 37°C in 5% (v/v) CO_2_ with CellTracker Red CMPTX dye (12.5 μM; Invitrogen, Paisley, Scotland) for 45 min to stain the cytoplasm. Cells were then fixed with 4% (w/v) paraformaldehyde. The nuclei were stained by incubation with 4’6-diamidino- 2-phenylindole dihydrochloride (DAPI; 250 μM) for 3 min. Cells were viewed on a confocal microscope with Krypton-Argon laser fluorescence detector.

### Cell viability

Cell viability, as determined by metabolic activity, was performed using an MTT (3-(4,5-dimethylthiazol-2-yl)- 2,5-diphenyltetrazolium bromide) assay.

### Data analysis

Results are expressed as mean ± SEM. A Kruskal-Wallis test or Dunnett's test was used for multi-group comparisons as appropriate. Differences between the effects of dexamethasone or formoterol or dexamethasone and formoterol treatment were analysed using Wilcoxon paired t-test. A Mann-Whitney test was also used where appropriate. Correlations were determined using Spearman rank correlation coefficient. A p value of <0.05 was considered significant.

## Results

### Participant characteristics

Severe asthmatics had lower FEV_1_ (% predicted) and FVC (% predicted) compared to both non-severe asthmatics and normal subjects (Table 1). They were also on higher doses of inhaled corticosteroids and reported a greater frequency of exacerbations. Of 14 patients with severe asthma recruited to the MDM study (Table 1), 10 were on daily oral prednisolone. In the severe asthmatics recruited to the AM study (Table 2), there was a trend towards higher eosinophil and neutrophils counts in BAL compared to both healthy and non-severe asthmatic subjects. Three subjects had studies performed on AMs and MDMs.

**Table 1:** Characteristics of subjects in monocyte-derived macrophage study

	**Normal**	**Non-severe asthma**	**Severe asthma**
Number	14	14	14
Sex (F:M)	6:8	8:6	9:5
Age (years)	39.6 ± 2.7	45.9 ± 3.6	48.6 ± 2.9
Atopy^#^	2/14	10/14	14/14
Duration of asthma (years)	0	33.1 ± 4.0	37.9 ± 4.6
Exacerbations in past year	0	1.57 ± 0.48	3.78 ± 0.26***
FEV_1_ (% predicted)	98.31 ± 3.28	83.40 ± 3.41	68.24 ± 4.29**
FVC (% predicted)	97.00 ± 3.16	95.51 ± 3.70	81.99 ± 2.65**
Oral prednisolone (mg/d)	0	0	14.50 ± 3.0*** (n = 10)
Inhaled cortico-steroid, BDP equivalent (μg/day)	0	971 ± 203 (n = 11)	2257 ± 321** (n = 14)

*Abbreviations*: *BDP* Beclomethasone dipropionate, *FEV*_*1*_ forced expiratory volume in 1 sec, *F* Female, *FVC* forced vital capacity, *M* Male.

^#^Atopy defined as positive skin prick test to one or more aeroallergens.

Data shown as mean ± SEM. **p < 0.01, ***p < 0.001 compared to non-severe asthma.

**Table 2:** Characteristics of subjects in the alveolar macrophage study

	**Normal**	**Non-severe asthma**	**Severe asthma**
n	7	6	8
Sex (F:M)	2:5	3:3	5:3
Age (years)	39 ± 5.6	34.7 ± 6.0	43.75 ± 29
Atopy^#^	1/7	6/6	6/8
Duration of asthma (years)	0	25.2 ± 5.9	35.8 ± 5.2
Exacerbation in past year	0	0.33 ± 0.33	3.25 ± 0.41***
FEV_1_ (% predicted)	94.71 ± 15.7	83.08 ± 4.25	79.69 ± 4.95
FVC (% predicted)	95.27 ± 14.65	97.43 ± 7.55	89.11 ± 4.35
Oral prednisolone (mg/day)	0	0	8.13 ± 4.8 (n = 3)
Inhaled corticosteroid BDP equivalent (μg/day)	0	600 ± 263	2200 ± 414*
Total BAL count (×10^6^)	10.7 ± 3.8	8.67 ± 3	7.3 ± 1.9
Cells/ml (×10^3^)	85 ± 24.4	74 ± 24.3	63.8 ± 18.9
Lymphocyte (%)	18.6 ± 7	7.9 ± 1.4	5.12 ± 1.6
Eosinophil (%)	0.6 ± 0.2	2.04 ± 1.2	4.14 ± 2.4
Macrophage (%)	76.6 ± 7.7	88.2 ± 1.7	84.6 ± 12.4
Neutrophil (%)	2.97 ± 1.2	1.37 ± 0.4	5.6 ± 2

*Abbreviations*: *BAL* Bronchoalveolar lavage, *BDP* Beclomethasone dipropionate, *F* Female, *FEV*_*1*_ forced expiratory volume in 1 sec, *FVC* forced vital capacity, *M* male.

^#^Atopy defined as positive skin prick test to one or more common aeroallergens.

Data shown as mean ± SEM. *p < 0.05, ***p < 0.001 compared to non-severe asthma.

### Phagocytosis by MDMs

**Figure 1:**
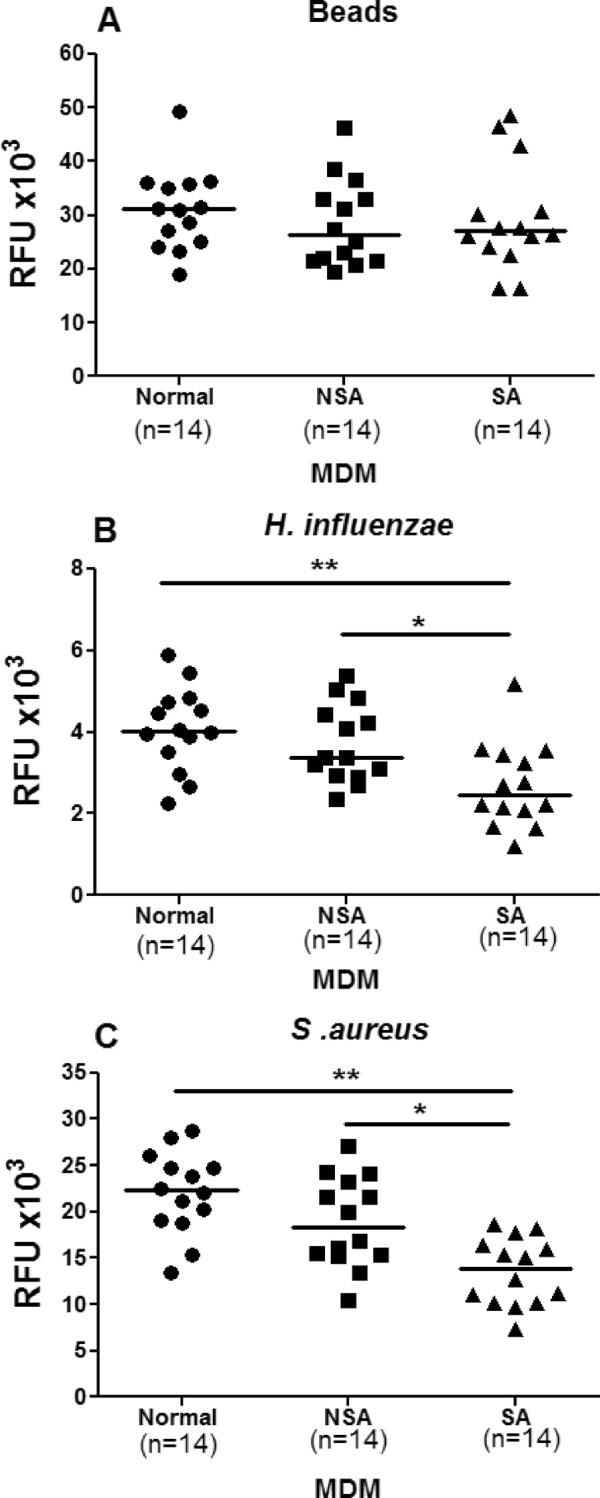
**Phagocytosis of fluorescently-labelled beads (Panel A), *H. influenzae *(Panel B) or *S. aureus *(Panel C) by monocyte-derived macrophages (MDM).** MDM were obtained from normal subjects (●, n = 14) and patients with non-severe asthma (NSA; ■, n = 14) or severe asthma (SA; ▲, n = 14). Phagocytosis was reported as relative fluorescence unit (RFU). Data are presented as dot plots and medians. A Kruskal-Wallis test followed by Dunnett's post-hoc test was used for multi-group comparisons, where *p < 0.05 and **p < 0.01.

Having established the assay conditions, MDMs from normal subjects and patients with non-severe and severe asthma were exposed to fluorescently-labeled beads or bacteria. There was no difference in phagocytosis of beads between the groups (Figure 1A). However, there was impaired phagocytosis of fluorescently-labelled *H. influenzae* by MDMs from severe asthmatics compared to cells from non-severe asthmatic patients (p < 0.05) and normal subjects (p < 0.01; Figure 1B). MDM phagocytosis of fluorescently-labelled *S. aureus* was also impaired in severe asthma compared to non-severe asthma (p < 0.01) and normal subjects (p < 0.001). There was no difference in the ability of MDMs from non-severe asthmatics to phagocytose either bacterial species compared to normal subjects.

### Phagocytosis by alveolar macrophages

**Figure 2:**
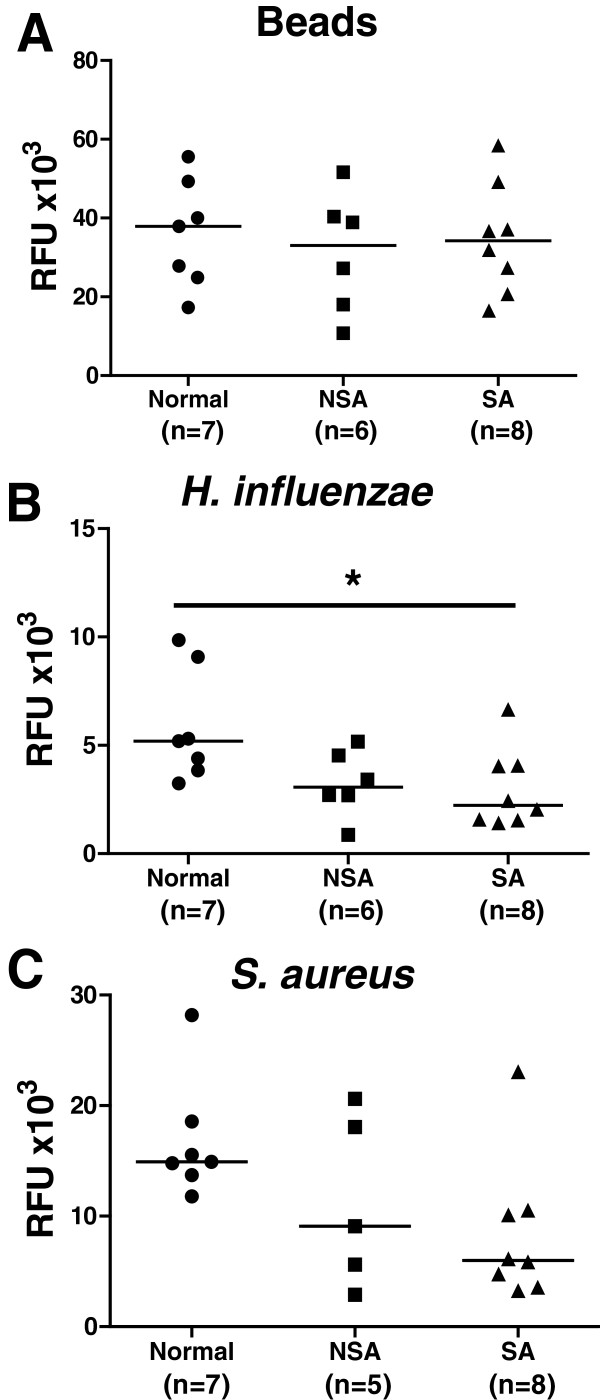
**Phagocytosis of beads, *H. influenzae *or *S. aureus *by alveolar macrophages.** Alveolar macrophages from normal subjects (●, n = 7) and patients with non-severe asthma (NSA; ■, n = 6) or severe asthma (SA; ▲, n = 8) were obtained by bronchoalveolar lavage. Alveolar macrophages were exposed to fluorescently-labeled beads **(Panel A)** or *H. influenzae* or *S. aureus***(Panels B and C)** for 4 h and phagocytosis reported as relative fluorescence units (RFU). Data are presented as dot-plots and medians. A Kruskal-Wallis test by Dunnett's post-hoc test was used for multi-group comparisons where *p < 0.05. Direct comparison between normal subjects and SA for *S.aureus* (C) was performed using a Mann-Whitney t-test where ^$^p < 0.01.

AMs from all subject groups had a similar capacity to phagocytose beads (Figure 2A). AMs from severe asthmatic patients exhibited a reduced capacity to phagocytose bacteria similar to that observed in MDMs (Figure 2B, 2C). There was impaired phagocytosis of fluorescently-labelled *H. influenzae* in severe asthma compared to normal subjects (Figure 2B; p < 0.05 (Kruskal-Wallis, post-hoc test)); for *S. aureus*, this impairment did not reach significance. However, there was a significant difference between the phagocytic response to *S. aureus* when healthy subjects were directly compared to severe asthmatics (p < 0.01; Mann-Whitney).

### Phagocytosis, FEV_1_ and eosinophilia

**Figure 3:**
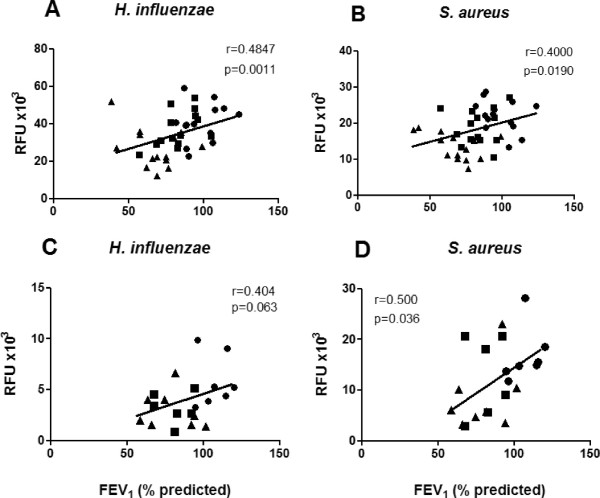
**Relationship between phagocytosis and lung function parameters.** MDM were derived from normal subjects (●, n = 14), non-severe (■, n = 14) and severe asthmatics (▲, n = 14) and exposed to either fluorescently-labeled *H. influenzae***(Panel A)** or *S. aureus***(Panel B)**. Alveolar macrophages from normal subjects (●, n = 7) and patients with non-severe asthma (NSA; ■, n = 6) or severe asthma (SA; ▲, n = 8) were exposed to either fluorescently-labeled *H. influenzae***(Panel C)** or *S. aureus***(Panel D)**. Correlations were determined using Spearman rank correlation coefficient.

Phagocytosis by MDMs of both *H. influenzae* (r = 0.48, p = 0.001, Figure 3A) and *S. aureus* (r = 0.40, p = 0.019, Figure 3B) correlated with FEV_1_ (% predicted). Phagocytosis by AMs of *H. influenzae* did not significantly correlate with FEV_1_ (% predicted) (r = 0.4, p = 0.06, Figure 3C) but phagocytosis of *S. aureus* (r = 0.50, p = 0.036, Figure 3D) did positively correlate with FEV_1_ (% predicted). For both MDMs and AMs, neither FEV_1_/FVC ratio nor age correlated with any of the phagocytic responses (data not shown). For AMs, there was an inverse correlation between the phagocytic response to *S. aureus* (but not to *H. influenzae*) and the % eosinophil in BAL fluid (r = -0.57; p < 0.01; Additional file 1: Figure S2).

### Internalisation of *S. aureus* by MDM

**Figure 4:**
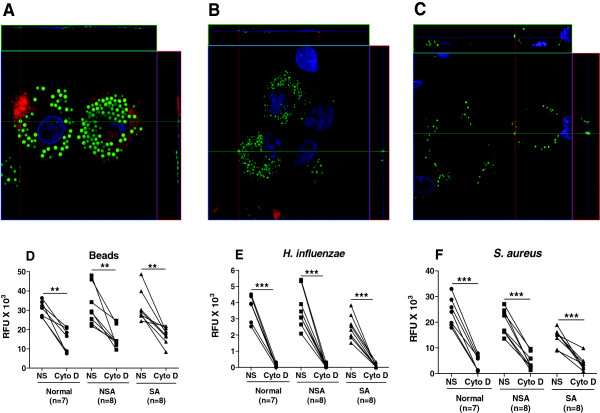
**Confocal microscopy of fluorescently-labeled beads or bacteria by MDM and effect of cytochalasin D. Panels A-C:** MDM were derived from a patient with non-severe asthma and exposed to either fluorescently-labelled beads **(Panel A)** or *S. aureus* for 4 h (green) **(Panel B)**. **Panel C** shows lack of phagocytosis of *S.aureus* in the presence of cytochalasin D. Cell cytoplasm was stained with Cell Tracker Red and the nuclei with DAPI (blue). **Panels D-F:** MDMs were derived from normal subjects (●), non-severe asthmatics (NSA; ■) or severe asthmatics (SA; ▲) and pretreated with cytochalasin D (cyto D; 5 μg/ml) prior to incubation with beads **(Panel D)**, *H. i influenzae***(Panel E)** or *S. aureus***(Panel F)** for 4 h. Phagocytosis was measured by fluorimetry and reported as relative fluorescence units (RFU). NS = not stimulated. A paired t-test was used to determine the effect of Cytochalasin D on phagocytosis. **p < 0.01, ***p < 0.001.

Confocal microscopy was used to confirm that particles and bacteria were being internalized (Figure 4) and showed uptake of both beads and *S. aureus* within MDMs (Figure 4A and B). In order to further confirm the process of internalization, MDMs were exposed to cytochalasin D (5 μg/ml), an inhibitor of actin filament polymerization, prior to incubation with *S. aureus* (Figure 4C). This led to an inhibition of phagocytosis with *S. aureus* unable to enter the cells, localizing to the outer cell membrane of MDMs. Having established that cytochalasin D prevented phagocytosis, we determined whether the fluorimetric plate reader assay could distinguish between intra-cellular bacteria (phagocytosis) and outer membrane-associated bacteria. Phagocytosis experiments were then performed in the absence and presence of cytochalasin D. Phagocytosis of beads, *H. influenzae* and *S. aureus* was suppressed in MDMs from all patient groups as indicated by the difference in fluorescence units (Figure 4D-F). There was a significant difference for all groups and particles between non- stimulated and Cyto D treated cells (Figure 4D-F).

### Effect of dexamethasone on phagocytosis

**Figure 5:**
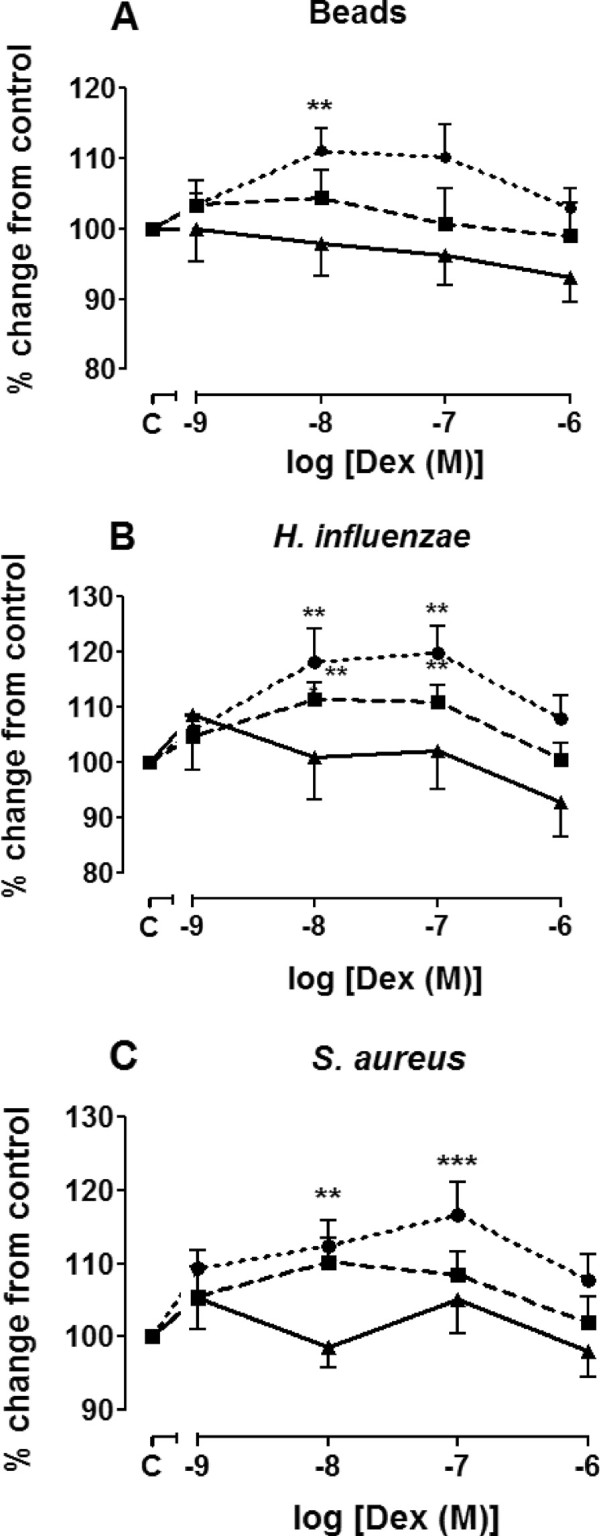
**Effect of dexamethasone on phagocytosis by MDM.** MDM from normal subjects (●, n = 14), non-severe asthmatics (■, n = 14) and severe asthmatics (▲, n = 14) were pre-treated with dexamethasone for 1 h prior to exposure to fluorescently-labelled beads **(Panel A)**, *H. influenzae***(Panel B)** or *S.aureus***(Panel C)** for 4 h. Phagocytosis was measured by fluorimetry and presented as mean ± SEM. A Mann-Whitney test was used to compare the effect of given concentration of dexamethasone with that of untreated samples. **p < 0.01 and ***p < 0.001 compared to untreated cells.

In order to investigate whether corticosteroids could influence the phagocytic response, MDMs isolated from normal subjects, and non-severe and asthmatics were exposed to dexamethasone prior to addition of beads or bacteria. Dexamethasone improved phagocytosis of beads and bacteria by MDM from normal subjects and non-severe asthmatics to a small extent (~10%) at low concentrations, reaching significance at 10^-8^ M, and also at 10^-7^ M for beads only, but this effect was not seen at higher concentrations of dexamethasone (Figure 5). Dexamethasone did not change the phagocytic response of MDMs obtained from patients with severe asthma. Dexamethasone did not affect cell metabolic activity as measured by MTT assay (Additional file 1: Figure S1A).

### Effect of formoterol alone or in combination with dexamethasone

**Figure 6:**
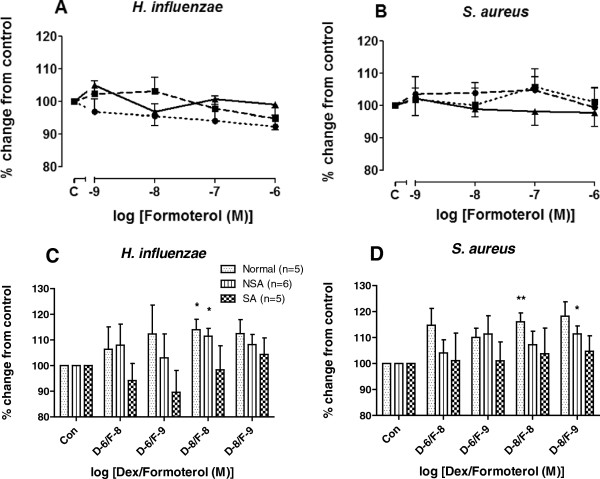
**Effect of formoterol alone or in combination with dexamethasone on phagocytosis.** MDM were derived from from normal subjects (●, n = 5), non-severe asthmatics (■, n = 6) and severe asthmatics (▲, n = 5) were pre-treated with formoterol in the absence or presence of dexamethasone for 1 h prior to exposure to *H. influenzae***(Panels A and C)** or *S. aureus***(Panels B and D)** for 4 h. Phagocytosis was measured by fluorimetry and data presented as mean ± SEM. Differences between the effects of dexamethasone or formoterol or dexamethasone and formoterol treatment were analysed using Wilcoxon paired t-test, where *p < 0.05 and **p < 0.01 compared to untreated cells. D = dexamethasone, F = formoterol.

The effect of the long-acting inhaled β_2-_agonist, formoterol, and the combination of dexamethasone and formoterol on phagocytosis of MDMs was investigated. Formoterol alone had no effect on phagocytosis of bacteria by MDMs in any of the subject groups (Figure 6A-B). The combination of formoterol with dexamethasone at 10^-8^ M each caused a small improvement of phagocytosis of *H. influenzae* by MDMs from normal subjects and non-severe asthmatics (p < 0.05, Figure 6C), but this was not seen in MDMs from severe asthmatic subjects (Figure 6C and 6D). For phagocytosis of *S. aureus* by MDMs, there was also an improvement with dexamethasone and formoterol at 10^-8^ M for normal individuals (p < 0.01, Figure 6D) and for non-severe asthmatics with dexamethasone and formoterol at 10^-8^ and 10^-9^ M (p < 0.05, Figure 6D). Formoterol did not affect cell metabolic activity as measured by MTT assay (Additional file 1: Figure S1B).

## Discussion

We investigated whether macrophage clearance of bacterial species associated with asthmatic airways were altered in asthma. We showed that there was a reduction in phagocytosis of fluorescently-labelled *H. influenzae* and *S. aureus* by MDMs from patients with severe asthma compared with MDMs from healthy controls; however this defect was not apparent in MDMs from non-severe asthmatics. We also showed that alveolar macrophages from severe asthma patients exhibited compromised phagocytic capacities for these bacterial species. However, the phagocytic response of these macrophages and MDMs to polystyrene beads was not altered in cells from patients with asthma, indicating that the defect was related specifically to these bacteria. These results are an extension of previous observations in children, where phagocytosis of inactivated *S. aureus* was impaired in alveolar macrophages from patients with severe asthma and to a lesser extent in those with moderate asthma [12]. This observation may underlie the increased risk of bacterial infections that has been described in patients with stable severe asthma [8,20]. In addition, this defect in bacterial phagocytosis may explain partly the presence of the altered microbiome that has been described in the airways of patients with asthma compared with healthy subjects [9]. A similar defect in the phagocytosis of bacterial pathogens by MDMs and AMs from patients with COPD has also been reported [15], also in association with an altered microbiome in the airways of patients with COPD [9,21].

MDMs and macrophages obtained from non-severe asthmatic patients did not show reduced phagocytosis of *H. influenzae* and *S. aureus* compared to normal subjects suggesting that the phagocytic defect was associated with more severe asthma. Severe asthma exhibits a number of similarities with COPD such as the presence of airway neutrophilia [22]. Our results are in line with a recent study showing that macrophages isolated from induced sputum from asthmatic subjects with increased neutrophil counts in the sputum were also less able to phagocytose apoptotic bronchial epithelial cells [23], indicating a defect in the process of efferocytosis in the airways of patients with asthma. The defect in phagocytosis of bacteria could lead to a persistence of airway bacteria resulting in airway neutrophilia through the release of neutrophilic chemokines such as CXCL8 and possibly to worsening of asthma in terms of severity of symptoms and recurrence of exacerbations. Recent work has indicated that *in-vitro* infection of lung tissue macrophages with rhinovirus resulted in an impairment of the phagocytosis of bacteria by alveolar macrophages [24]. This mechanism of defective bacterial phagocytosis by the presence of rhinovirus may be relevant to acute severe asthma, where a high percentage of positive bacterial cultures from sputum samples has also been reported [25].

The cellular and molecular mechanisms for this phagocytic defect of macrophages from patients with severe asthma are unclear. Macrophages are important for both innate and acquired immunity in the respiratory tract, and have a pivotal role in lung defense against viruses, bacteria and fungi [26]. The findings of this study indicate that, in severe asthma, an impairment of the ability of alveolar macrophages to phagocytose bacteria could lead to prolonged persistence of bacteria in the airways and lungs, which could contribute to the worsening of asthma control. In a previous study, we showed that the release of pro-inflammatory cytokines from peripheral blood mononuclear cells and alveolar macrophages from patients with asthma, stimulated with lipopolysacharide, was not different from that of patients with non-severe asthma [27,28], indicating that the inflammatory response to one constituent of gram-negative bacteria is not impaired in severe asthma. However, the response of these cells to whole bacteria is not known. Reactive oxygen species production by alveolar macrophages is required for bacterial killing [29], and is also important for phagocytosis. In mice lacking the antioxidant enzyme extracellular superoxide dismutase [30], macrophage phagocytosis of bacteria was impaired, suggesting that removal of reactive oxygen species within alveolar macrophages is required for normal phagocytosis.

There was a positive correlation between airflow obstruction as measured by FEV_1_ and phagocytosis of bacteria, either *S. aureus* or *H. influenzae*, by alveolar macrophages and MDMs. The link between the reduced bacterial phagocytosis and airflow obstruction remains unclear. Airflow obstruction in severe asthma has been associated with eosinophilic inflammation, duration of asthma and thickening of the airways as measured by high resolution computed tomography [8]. It is possible that the link may lie with airway wall remodelling, as this has been associated with the presence of chronic airflow obstruction. The negative correlation between the percentage of eosinophils in bronchoalveolar lavage fluid and the phagocytic response to *S. aureus* of macrophages from asthma patients also support a potential role for eosinophilic inflammation. Eosinophlic inflammation is an important source of oxidative stress in asthma [31], and oxidative stress has been linked to defective phagocytosis of bacteria [32].

Inhaled corticosteroids and β-agonists remain the mainstay of treatment of many patients with asthma [33]. Patients with more severe asthma are usually treated with high doses of these agents. In addition, the majority of the patients with severe asthma in our study were also on oral corticosteroid therapy. However, many patients with severe asthma may not achieve control and this lack of therapeutic response to corticosteroids has been attributed to the development of corticosteroid insensitivity [27,28]. Oxidative stress pathways [34] and activation of mitogen-activated protein kinases have been implicated as mechanisms of corticosteroid insensitivity in severe asthma [27,35]. Bacterial interactions with epithelial cells and macrophages can activate these pathways and therefore could underlie corticosteroid insensitivity.

Neither dexamethasone nor formoterol had any deleterious effects on the phagocytic response in MDM from any of the subject groups. Dexamethasone alone or combined with formoterol slightly improved the phagocytic response of MDM for both beads and bacteria obtained from healthy subjects but not from those with asthma. The significance of this small increase is unlikely to be clinically relevant. There was also no effect of the phagocytic response towards either *H. influenzae* or *S. aureus* in severe asthma by dexamethasone or formoterol, which contrasts to the increase in phagocytosis of bacteria by MDMs stimulated by budesonide previously reported in COPD patients [15]. However, in another study, budesonide decreased phagocytosis in alveolar macrophages from healthy never-smoked subjects by decreasing the number of cells with the ability to phagocytose as well as decreasing phagocytic capacity of these cells [36]. These short-term studies will not categorically exclude the possibility that long-term usage of corticosteroids and long-acting β-agonists in severe asthma may have a detrimental effect on macrophage phagocytosis.

A possible shortcoming of the study could be the lower number of subjects recruited to the AM study as a result of the inherent problems associated with collecting BAL samples from severe asthmatics. However, these studies performed in AM were proof of principle studies to confirm, *ex vivo*, the findings of the MDM study. Moreover, although there are studies showing that macrolides [37] and antifungal agents [38] can improve the phagocytic capabilities of macrophages, our results show that in the presence of antifungal and/or antibacterial agents, AMs and MDMs from patients with asthma continue to display an inherent impaired phagocytic response compared to those from control subjects

In summary, MDMs and AMs from severe asthma patients demonstrate reduced phagocytosis of fluorescently-labelled *H. influenzae* and *S. aureus*. This may contribute to the bacterial colonisation of the lower airways and to the propensity for bacterial exacerbations in severe asthma. This defect is unlikely to result from an acute response to corticosteroid or β-adrenergic therapy. Further work is needed to determine the cellular and molecular basis of this phagocytic defect for bacteria.

## Conclusions

Persistence of bacteria in the lower airways may result partly from a reduced phagocytic capacity of macrophages for bacteria. This may contribute to increased exacerbations, airway colonization and persistence of inflammation.

## Abbreviations

AM: Alveolar macrophage; BAL: Bronchoalveolar lavage; COPD: Chronic obstructive pulmonary disease; FEV_1_: Forced expiratory volume in 1 sec; FVC: Forced vital capacity; *H. influenzae*: *Haemophilus influenzae*; MDM: Monocyte derived macrophage; PBMC: Perpheral blood mononuclear cells; PC_20_: Provocative concentration of metacholine causing a 20% fall in FEV_1_; *S. aureus*: *Staphylococcus aureus.*

## Competing interests

PKB has received project grant funding from GlaxoSmithKline. PJB has received project grant funding from GlaxoSmithKline and Astra-Zeneca. KFC has received project grant funding from GlaxoSmithKline and Pfizer. LED has received project grant funding Astra-Zeneca and Pfizer. The other authors have no competing interests.

## Authors’ contributions

ZL participated in the design of the study, carried out all assays on MDMs, performed the statistical analysis and drafted the manuscript. QZ participated in the design of the study, and performed assays on AMs. CMRT participated in the design of the study and generated labelled *Haemophilus influenzae.* KR performed assays on AMs. DG performed bronchoalveolar lavage on subjects for the AM study. PJB participated in the design and coordination of the study. LED participated in the design and coordination of the study and critically revised the manuscript. PKB and KFC conceived the study, participated in the design and coordination of the study and wrote the manuscript. All authors read and approved the final manuscript.

## Supplementary Material

Additional file 1: Figure S1 Effect of dexamethasone or formoterol on metabolic activity of MDM. MDM from normal subjects, non-severe asthmatics and severe asthmatics were treated with dexamethasone (A) or formoterol (B) for 1h and cells assayed for metabolic activity using MTT assay. **Figure S2.** Relationship between phagocytosis of *S. aureus* by AM and % eosinophils in BAL. Alveolar macrophages from normal subjects (●, n = 7) and patients with non-severe asthma (■, n = 6) or severe asthma (▲, n = 8) were exposed to fluorescently-labelled *S. aureus*. Correlations were determined using Spearman’s rank correlation coefficient. Click here for file
