# Supplementary material for: Impaired macrophage phagocytosis of bacteria in severe asthma
Source: Respir Res. 2014 Jun 27;15(1):72. doi: 10.1186/1465-9921-15-72 (PMC4086996; doi:10.1186/1465-9921-15-72)
Supplement: Additional file 1: Figure S1 — Effect of dexamethasone or formoterol on metabolic activity of MDM. MDM from normal subjects, non-severe asthmatics and severe asthmatics were treated with dexamethasone (A) or formoterol (B) for 1h and cells assayed for metabolic activity using MTT assay. Figure S2. Relationship between phagocytosis of S. aureus by AM and % eosinophils in BAL. Alveolar macrophages from normal subjects (●, n = 7) and patients with non-severe asthma (■, n = 6) or severe asthma (▲, n = 8) were exposed to fluorescently-labelled S. aureus. Correlations were determined using Spearman’s rank correlation coefficient. [file 1465-9921-15-72-S1.docx]

**Additional file**

Impaired macrophage phagocytosis of bacteria in severe asthma

Zhike Liang^1 2^, Qingling Zhang^1 2^, Catherine M R Thomas^1^, Kirandeep K Chana^1^, David Gibeon^1^, Peter J Barnes^1^, Kian Fan Chung^1^, Pankaj K Bhavsar^1^ and Louise E Donnelly^1^.

Figure S1

**Figure S1: Effect of dexamethasone or formoterol on metabolic activity of MDM.** MDM from normal subjects, non-severe asthmatics and severe asthmatics were treated with dexamethasone (**A**) or formoterol (**B**) for 1h and cells assayed for metabolic activity using MTT assay.

Figure S2

**Figure S2**. **Relationship between phagocytosis of *S. aureus* by AM and % eosinophils in BAL.** Alveolar macrophages from normal subjects (●, n=7) and patients with non-severe asthma (■, n=6) or severe asthma (▲, n=8) were exposed to fluorescently-labelled *S. aureus*. Correlations were determined using Spearman’s rank correlation coefficient.
